# Access to information and use of adolescent sexual reproductive health services: Qualitative exploration of barriers and facilitators in Kisumu and Kakamega, Kenya

**DOI:** 10.1371/journal.pone.0241985

**Published:** 2020-11-12

**Authors:** Lilian Mutea, Susan Ontiri, Francis Kadiri, Kristien Michielesen, Peter Gichangi

**Affiliations:** 1 U.S. Agency for International Development Kenya and East Africa, Nairobi, Kenya; 2 Faculty of Medicine and Health Sciences, Ghent University, Ghent, Belgium; 3 Jhpiego, John Hopkins University Affiliate, Nairobi, Kenya; 4 International Centre for Reproductive Health, Department of Public Health and Primary Care, Faculty of Medicine and Health Sciences, Ghent University, Ghent, Belgium; University of Sydney, AUSTRALIA

## Abstract

**Background:**

Kenya has a high prevalence of adolescent pregnancy and low access to and use of adolescent sexual reproductive health services. Despite the enactment of evidence-based policies to address this problem, adolescents continue to face health problems and barriers to adolescent sexual reproductive health information and services.

**Main objective:**

This study describes barriers to and facilitators of access to adolescent sexual and reproductive health services in Kisumu and Kakamega counties, Kenya.

**Methodology:**

We used a qualitative design. Through 61 data collection sessions, 113 participants were engaged in key informant interviews, in-depth interviews, and/or focus group discussions. Trained Research Assistants (RAs) engaged adolescents, health care workers, teachers, county leaders, and community representatives. Data were captured using audio recorders and field notes. Socio-demographic data were analyzed for descriptive statistics, while audio recordings were transcribed, translated, and coded. Thematic analysis was done with NVivo.

**Results:**

Findings show that the barriers of access to sexual reproductive health services and information were negative health workers’ attitudes, distance to the health facility, unaffordable cost of services, negative social cultural influences, lack of privacy and confidentiality. Facilitators to adolescent sexual reproductive health services were few and included getting priority for school going adolescents and enabling environment for partnerships on adolescent health issues.

**Conclusions:**

Adolescents in Kakamega and Kisumu face a myriad of barriers when seeking sexual reproductive health information and/or health services. We recommend that counties sensitize all stakeholders on adolescent sexual reproductive health problems, and support development of multi-sectoral, sustainable solutions to adolescent health needs.

## Introduction

Adolescents comprise 24.5% of Kenya’s 47.6 million total population [1]. This segment of the population is at high risk of sexually transmitted infections (STIs), including HIV/AIDS while female adolescents face the additional risk of early pregnancy, unsafe abortion and female genital mutilation [2]. In Kenya, the age of sexual debut is low; 47% of young women and 55% of young men between the ages of 18–24 years reported sexual intercourse before the age of 18 years [3]. Consequently, pregnancy among adolescents aged 15–19 years continues to be a significant problem in Kenya, with a teenage pregnancy rate of 18% and an adolescent birth rate of 96 per 1,000 women [3]. Studies have documented that adolescent pregnancy, whether intended or unintended, increases the risk of maternal mortality and morbidity, including complications of unsafe abortion, prolonged labor and delivery, and sepsis during the postnatal period [4]. Furthermore, more than half (51%) of all new HIV infections in Kenya in 2015 occurred among adolescents and young people (aged 15–24 years), with young women accounting for 33% of the total number of new infections [5]. Studies have shown that women who become mothers in their teens are more likely to drop out of school and have reduced career progression and economic empowerment, perpetuating the cycle of poverty [6]. Adolescents also experience a high rate of violence, with the 2019 report of violence against children indicating that sexual violence was experienced by 15.6% of females and 6.4% of males before age 18 years in Kenya [7].

To address these challenges, Kenya developed a National Adolescent Sexual and Reproductive Health policy to guide implementation of interventions aimed at assisting the country to achieve the development goals [8]. Despite this legal framework, implementation of adolescent sexual and reproductive health (ASRH) services has been weak, and, consequently, barriers to access and use of SRH services among adolescents and youth continue to exist [9]. This may, for instance, explain the low (36.8%) contraceptive prevalence rate among married adolescent females (15–19 years), and among sexually active unmarried adolescents (49.3%); and the high unmet need for contraceptives, estimated at 23% as compared to 18% for older women [3]. Constitutionally, abortion is illegal unless, in the opinion of a qualified medical practitioner, the health or the life of the woman is at risk or unless permitted by any other law [10]. Despite this, unsafe abortion is rife among adolescents and youth and underreported [11]. The constitution also stipulates 18 years as the legal age of marriage and consent to sex [10]. However, 23% of Kenyan girls are married before their 18th birthday and 4% are married before the age of 15 [12].

To operationalize the 2015 National Adolescent Sexual and Reproductive Health Policy, the Ministry of Health allows health workers to provide SRH services to adolescents who are minor but are considered mature due to their experience, education, training/conduct, marital status, and or involvement in making important decisions in their lives [8]. Additionally, Kenya’s national guidelines on family planning recommend that mature minors be allowed to consent to receive health services. Mature minor are described as those who are married, have children, STIs or symptoms of STIs, are pregnant or have ever been pregnant [13]. While use of ASRH services, such as contraception is a key approach to prevent early pregnancy, all adolescents in lower- and middle-income countries, especially unmarried ones, face a number of barriers at the individual level and in their environment [14]. We aimed to identify these barriers for stakeholders to develop context-specific solutions to address the problems of poor access and low use ASRH services. This paper details an exploratory study of the barriers and facilitators to access SRH services by adolescents in Kakamega and Kisumu counties in Kenya. The work reported herein emanates from the ASRH interventions that were led by the U.S. Agency for international Development-funded Afya Halisi (“real health” in Swahili) project in Kenya. The ASRH component seeks to improve access and use of quality ASRH services and reduce the burden of adolescent pregnancy.

## Materials and methods

### Study site and design

This qualitative study was a formative phase of a larger study, whose findings were used as a baseline for an ASRH intervention [15], that was carried out in two wards that were purposively selected: Kobura ward within Nyando sub-county (Kisumu county) and Kholera ward within Matungu sub-county (Kakamega County). These two wards and sub-counties were among those with the highest burden of adolescent pregnancy and low use of ASRH services within the two counties. The study team conducted key informant interviews (KIIs), in-depth interviews (IDIs), and focus group discussions (FGDs). RAs conducted IDIs among teachers, health care workers (HCWs), community representatives, and adolescents. FGDs were conducted with adolescents only, as they were the main study informants and it was important to get their perspective on the issues. KIIs were done with county leadership. Study enrolment was done through catchment health facilities, surrounding schools, and the community within the participants’ health facility catchment area—the surrounding geographic area from which a health facility attracts its clients.

### Sampling and recruitment strategy

The study team engaged five categories of KII informants: reproductive health coordinators; county directors of youth, education, and health; and high school head teachers. IDI were conducted with adolescents males and females, community representatives consisting of chief, village elders, parents, youth champions, religious leaders, police officers commanding the police division, and community health workers; primary and secondary school teachers; and HCWs from the following categories: public health facilities (sub-county hospitals, health centers, and dispensaries), private health facilities, and chemist/pharmacy attendants. FGDs with adolescents were organized, into female only, male only, and mixed male and female. During the design stage, the study leaders contacted county health authorities, described the study, and sought permission to carry out the study activities. Before implementation, the research teams approached community leaders and local administrative authorities and described the study to them after permission had been granted by the County Health Management Team (CHMT). The study team used a recruitment script to introduce the study and explain the purpose, study procedures, and rights of participants. Participants enrolled in the study at the nearest health facility, surrounding school, or community within the facility catchment area.

We used purposive sampling to select adolescents (male and female) aged 15–19 years. While the World Health Organization (WHO) classifies adolescents as those aged 10–19-year-old, our study focused on 15–19-year-olds because adolescents aged 10–14 are considered very young and interventions targeting this group were likely to be met with resistance by county governments and stakeholders. In Kenya, strong cultural and religious narratives have been instrumental in blocking implementation of ASRH interventions outlined in global commitments [16]. In addition, this study was a formative assessment for an intervention study designed to use a community, facility, and school approach and the school approach was designed to target secondary schools, which enroll adolescents above 14 years.

The study team mapped youth social networks based on the information shared by the local administration and the respective youth leaders were identified. The youth and church leaders collaborated with HCWs in the catchment area to select adolescent participants. Adolescents were selected based on their previous interaction with youth leaders and HCWs and those who met the inclusion criteria.

After participants were identified, RAs visited the adolescents to seek informed consent from those aged 18–19 years and from mature minors. They also sought parental permission and adolescent assent for adolescents aged 15–17 years. RAs provided a detailed explanation of the study to parents and, consequently, they provided their permission for their adolescent minors to participate. During the FGDs, RAs identified adolescents to take part in an IDI based on their engagement and ability to communicate verbally in the FGD. They were approached at the end of the session and asked to participate in an IDI, which provided an opportunity to further explain the process.

Health care providers were drawn from public and private health facilities, including pharmacies and local chemists who provide ASRH services in Kisumu and Kakamega counties. RAs obtained a list of all ASRH providers in the participating facilities from the sub-county reproductive health coordinator. The purposively selected providers were those with more years of experience providing ASRH services and were invited to participate through a phone call or a face-to-face meeting with the interviewer. If a provider declined, a new invitation was sent to a provider from the same or similar site. The IDI recruitment aimed to include a balance of cadres and genders of health providers, levels of care (dispensary, health centers, and sub-county hospital), and both the public and private sectors. Teachers were recruited from both primary and secondary schools in the study areas and were those responsible for guidance and counseling of adolescents. Investigators obtained a list of all guidance and counseling teachers from schools in the study area, then invited them individually to participate through a face-to-face meeting. If a teacher declined, a new invitation for a face-to-face meeting was sent to a teacher from the same or another participating school.

For the community representatives, investigators generated a list of key community influencers in the study area and selected men and women who had influence on ASRH services, including those who could pose barriers or create an enabling environment. The nine KIIs were similarly purposively identified and invited for face-to-face interviews.

All the study participants except for the adolescents were invited through telephone calls. For all study participants, we provided invitations that included details of the venue, date, and time for the activity. The study’s inclusion and exclusion criteria are presented in Table 1.

**Table 1:** Inclusion and exclusion criteria.

Participant category	Inclusion criteria	Exclusion criteria
Adolescents: FGD and IDI	15–19-year-old males and females who are residents of the selected study region Adolescents 18–19 years who provide informed consent 15–17 years* whose parents provide permission the minors provide assent Informed consent provided by mature minors aged 15–17 years	Adolescents in foster care Adolescents with mental disability
Community Representatives–IDI	Resident of the community Influencer in the community	
Health workers–IDI	ASRH provider in a public/private health facility or chemist	
Teachers–IDI	Responsible for guidance and counseling in a school within the study area	
Decision makers–KII	In leadership position on adolescent and youth matters in the county	

*A mature minor is defined as a “minor 15 years of age or older; living separate and apart from their parents or guardian, whether with or without the consent of a parent or guardian and regardless of the duration of the separate residence, and managing their own financial affairs, regardless of the source of income” [17]. In this study, adolescents aged 15–17 years who fit the definition of “mature minors” and could provide informed consent on their own behalf were included.

### Fieldwork team recruitment and training

A total of six qualitative RAs were recruited to help facilitate our study based on their prior understanding of ASRH issues and proven skills in conducting qualitative interviews. They had bachelor’s degree qualification in social sciences with significant experience conducting qualitative interviews and knew the local language (Dholuo and Lwanga). Two highly skilled and experienced supervisors with masters’ degree in public health managed the RAs and associated fieldwork activities. RAs attended a central, four-day training to receive background information on the study, familiarize themselves with the study tools and consent documents, and refresh their skills on effective interviewing. The training included overview lectures, small group discussions and role plays.

### Study variables

Our study used interview guides that had several domains which included issues affecting adolescents’ health, barriers and facilitators to access and use of SRH services by adolescents. Interview guides also included questions about myths, misconceptions, perceptions, and experiences participants had accessing and using SRH information and services; policies related to ASRH and questions about opportunities to increase access and use of ASRH services. The interview guides are provided in the S1 File.

### Data collection and management

The tools used for the study were translated into local languages and pretested in April 2019, prior to data collection. The pretesting was conducted in Nyakach sub-county and Matungu sub-county in Kisumu and Kakamega respectively. Challenges with pretest tools and methods were addressed prior to the data collection that was carried out in June 2019. During fieldwork, supervisors kept in constant communication with the RAs to ensure smooth flow of field activities. Prior to any data collection event, written consent (and assent as appropriate) was obtained. RAs obtained consent individually for each participant before the FGD and IDI, based on the age of the adolescent and according to study procedures. A moderator and a note taker facilitated each FGD session, while an interviewer conducted the IDIs and KIIs. All data collection sessions were conducted in safe spaces that were in use by the counties and the Afya Halisi project. These spaces were within easy reach of participants and located away from schools, health facilities, and homes. All sessions were captured through audio recording and note taking with consent of study participants.

### Data analysis

Data analysis was conducted in English. All data collection tools were developed in English and translated into Swahili (national language) and two local languages (Dholuo and Lwanga). All FGDs; IDIs and KII were conducted in English except the IDIs with community representatives, which were conducted in Swahili, Lwanga, and Dholuo. Following fieldwork, audio files from IDIs, FGDs, and KIIs were transcribed from the data files in the language in which they were recorded. Those in Swahili, Lwanga, or Dholuo were translated into English language and back translated to ensure that the original meaning was maintained, then entered into qualitative data software. Information from the handwritten notes was used to supplement information gaps from the audio-recorded transcripts. All 61 transcripts were analyzed with the use of NVivo 12 software. The study included six phases of thematic analysis as described by Braun and Clarke, which included: 1) becoming familiar with the data; 2) generating initial codes across the dataset and grouping coded data; 3) searching for themes by collating identified codes into themes and gathering data that was relevant to each theme; 4) reviewing themes and creating a map of the analysis; 5) defining and naming themes; and 6) producing an analysis report and selecting appropriate, vivid quotes in support of described themes [18]. We used both deductive and inductive analysis approaches. To ensure timely coding and validation of the coding frame, six coders were paired to facilitate cross-referencing of codes and data analysis.

**Fig 1:**
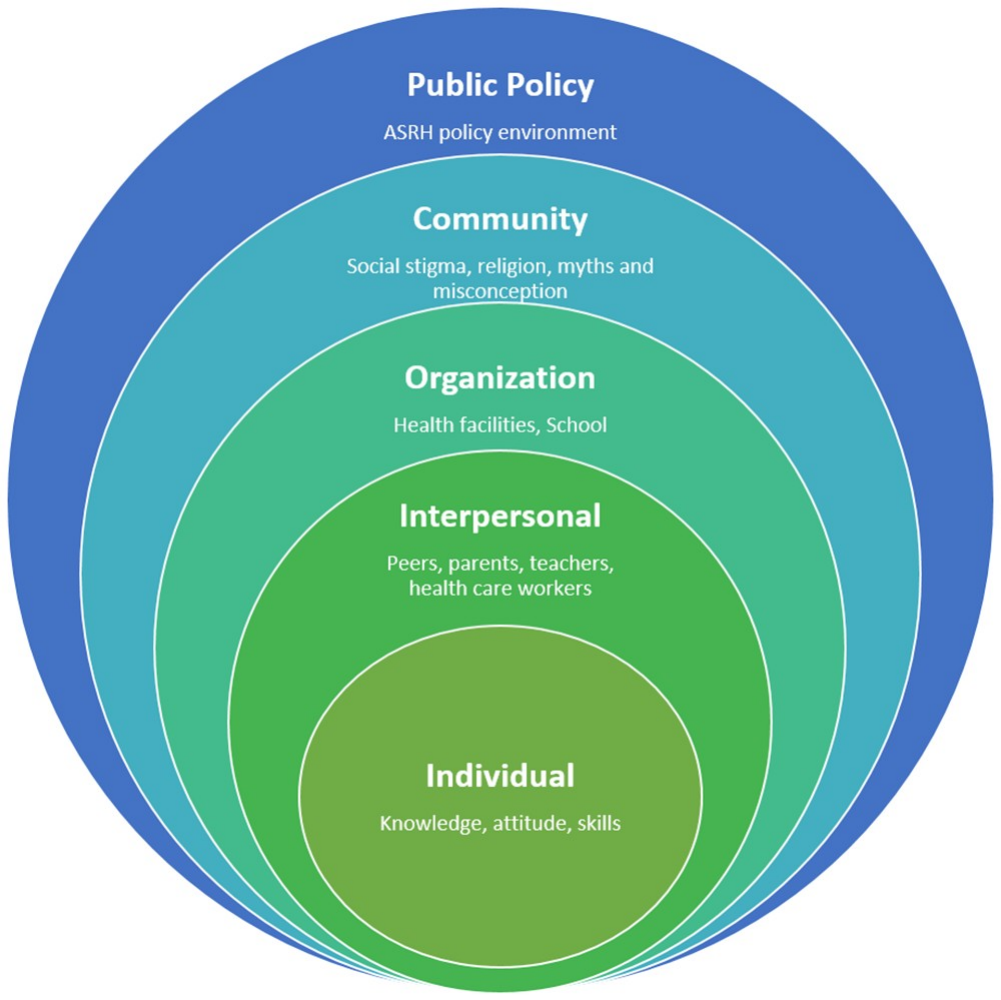
Ecological model showing levels of factors affecting access to ASRH information and services in Kisumu and Kakamega, Kenya.

In this study, we used the ecological framework to organize barriers and facilitators to ASRH services as this framework is useful in studying determinants of health behaviors, outcomes and outlines elements needed to provide an enabling environment that would enhance uptake of services [19]. The elements are at individual, relationship, organization, community, and policy levels (Fig 1).

### Ethical considerations

The Kenya Medical Research Institute research ethics reviews committee and the Johns Hopkins School of Public Health Institutional Review Board approved our study protocol. The research team had research ethics certification and adhered to the study protocol provisions, including ensuring informed consent, protection, and confidentiality of participants during data collection sessions, maintaining the confidentiality of all materials and information, limiting access to study information to only authorized personnel, and ensuring no identifying information on individual participants was included in the data analysis and reporting.

## Results

### Study participants’ demographics

The study conducted a total of 61 data collection sessions involving 113 participants; 30 were carried out in Kisumu and 31 in Kakamega. There was a nearly equal number of males and females engaged; 62% of participants were aged 15–19 years. All participants had some education, with 53% and 34% reporting secondary and tertiary levels, respectively. The demographic characteristics are summarized in Table 2.

**Table 2:** Demographic characteristics of participants in the ASRH study.

Variables		Adolescents (n = 70)	Community representatives* (n = 12)	Teachers (n = 14)	HCWs (n = 10)	County leaders* (n = 7)	Total (n = 113)	
							n	%
Type and number of Session		FGD-6	IDI-12	IDI-12	IDI-10	KII-7		
		IDI- 12		KII-2				
Sex	Female	33	2	11	7	3	56	49.6
	Male	37	10	3	3	4	57	50.4
Age (years)	15–19	70					70	62.0
	20–34		4	6	4		14	12.4
	35+		8	8	6	7	29	25.6
Education level	Primary	14	1				15	13.3
	Secondary	56	4				60	53.1
	Tertiary		7	14	10	7	38	33.6

*These include County Directors of Health, Youth and Education and Country Reproductive Health Coordinator.

### Common issues perceived to affect the health of adolescents in the community

Adolescents reported facing issues such as lack of access to contraceptives services and methods, particularly condoms. Participants mentioned child marriage was a challenge; as a result of poverty, parents offered their daughters for marriage in exchange for dowry. Adolescents, largely male also reported using bhang (marijuana) and alcohol due to pressure and social problems at home.

“When they are peer pressured, they can take alcohol and that can lead to drop out of school.”

(Kisumu, FGD 1, Girls)

“[One of the] things that causes rapes are the use of drugs. You may find that even an old grandmother may be raped by young people because drugs have destroyed their brains. Drugs use may also cause raping a child who is underage.”

(Kisumu, FGD 1, Boys)

Some adolescent females who were orphaned by HIV/AIDS and living with grandmothers were vulnerable due to poverty and lack of parental care; and were therefore susceptible to early pregnancies and school dropout. Adolescent females from poor families also reported consenting to sex in exchange for money and sanitary towels, but in the process were raped. In addition, Adolescents reported STIs, such as HIV, gonorrhea, and syphilis.

“You can get a young girl being convinced by an older person who has got money to indulge them in sex.”

(Kakamega, FGD 1, Girls)

#### Barriers to access and use of SRH information and services by adolescents

Barriers to use of ASRH information and services were reported at all five levels of the ecological model: individual, relationship, organization, community, and policy. Both adolescent and adults reported these barriers, which are described below.

*Individual-level barriers*. The most common barrier among adolescent participants that mentioned in all FGDs and IDIs was the lack of money to access services, including for transport to the health facility, consultation and medicine fees. This was especially important for management of STIs because an adolescent who has a referral needs fare and money to buy prescription drugs that are not available for free at the health facility.

“When someone wants to go to the hospital, they may lack the fare to take them there, or say it’s at night, getting a vehicle that takes them may be hard.”

(Kakamega, FGD 1, Girls)

The second most common barrier mentioned by majority of the adolescent respondents was the negative attitude of HCWs, which discouraged many adolescents from visiting health facilities even when they needed care.

“Some go to the hospital for the first time and find that the doctor is not friendly. They start fearing the doctor because he is harsh. They become afraid to go back for treatment.”

(Kakamega, FGD 1, Boys and Girls)

Some opted to travel to distant facilities to avoid encountering their social networks.

“Those who are in adolescence choose places where they are not known so that when they are diagnosed with certain illness, it will be a secret, and no one will know.”

(Kisumu, FGD 1, Girls)

Maybe when you go to the hospital, you know that somebody from your clan is there. So, you fear going there because they might find out and tell your parents.”

(Kakamega, FGD 1, Boys and Girls)

Some adolescents also had fears about disclosure of their SRH condition or concerns. They worried about the pain of injections and were apprehensive about side effects of contraceptives. Others were uninformed about SRH services and support available in their communities, while others believed in lay theories of illness, home births, and use of herbal medicine.

*Relationship-level barriers*. Some respondents reported that the absence of parental teachings on ASRH discouraged use of care services. SRH matters were rarely discussed at home; culturally, SRH is not a topic discussed much because adolescents are perceived as children and not as young adults with an active sexual life.

“I have parents at home, but maybe I’m afraid to talk to them. So, if someone else’s parent is discussing it, I could ask questions and they are ready to answer. Asking my father, he might have trouble talking to me about such things.”

(Kakamega, IDI, Boy 3)

“Parents do not want to give their children time to access this information, maybe because they feel it is not the right time…, but generally somebody who is 15 years, that one to me needs lots of counseling and guidance from both home and outside home.”

(Kisumu, IDI, Community Representative 4)

Almost all teachers interviewed reported filling a gap created when parents avoid addressing ASRH issues at home. A Kakamega teacher illustrated this well:

“To some, discussing sexual activities, they don’t have the guts. To some, they fear. They cannot even discuss with their kids, their families. So, we (teachers) are supposed to have a syllabus, an area where everybody just goes through that thing, they do an exam, they read it properly.… But it is not enough.”

(Kakamega, IDI, Teacher 2)

There are some parents who are very harsh, and we know them. We have interacted with them. Whenever you go to them with a particular issue, they will not respond to you well, so you will fear to go to them with the same issue next time, you just go talk to a friend.”

(Kisumu, FGD 3, Boys and Girls)

A few adolescents mentioned being uncomfortable with getting service from HCWs of the opposite gender. Many adolescents indicated HCWs did not observe confidentiality and privacy. Male adolescents from the two counties reported this barrier well:

“People might overhear… I felt that the place was not very private. Getting out of there was a big problem because I felt like everyone had heard what we talked about with the doctor.”

(Kakamega, IDI, Boy 3)

“… the doctor reads your name out loudly in public and what you are suffering from. This may make you even leave and go back home.”

(Kisumu, FGD 1, Boys)

*Organization-level barriers*. Were related to health facilities and schools; a long distance to SRH services, shortage of staff and long queues, health facility costs, and supply stock-outs were reported as organizational barriers to use of SRH care services.

“The number of people in the facility can make you stay there until evening.”

(Kakamega, FGD 1, Boys)

In addition, inflexible timings of SRH services, HCWs who were untrained on family planning methods, absence during services hours deterred use of services. All adolescents interviewed also wished for more youth-friendly spaces, which were not available in the health facilities.

“In this community I have not seen [youth-friendly spaces] because when you go to the hospitals, they just treat you like any regular patient.”

(*Kisumu, FGD* 2, Boys)

“Sometimes you got to the facility and the tell you that they don’t have medication or oral syringes.”

(Kakamega, IDI, Boy 3)

Adolescents who were in school encountered unique problems. They needed to confide in a teacher to get formal approval to go for care. Sometimes, they were afraid to disclose their condition and would, consequently, miss necessary health care. Where schools were far from health facilities, long distances coupled with unaccommodating timing of health services were a barrier. In addition, teachers were not fully trained to handle SRH matters and schools lacked well stocked dispensaries.

“Teachers are specialized in teaching but not generally in health, so as much we want to talk to them about such issues, what we give them is not enough.”

(Kisumu, IDI 4, Teacher)

“Whenever they are supposed to be going to take the medicine, their ARVs [antiretrovirals], they come and ask for permission.”

(Kisumu, IDI, Teacher 3)

*Community-level barriers*. Social stigma was reported as a barrier because adolescents did not want anyone, they knew to see them at the health facility receiving SRH services due to association of ASRH services with sexual activities, which were sometimes labeled as “bad manners.”

“Stigma, if you are seen going to the hospital, it’s like you’re engaging in sex. So, society will have a particular perception of you.”

(Kisumu, IDI, County leader 1)

“They [community members] will just see her and say, ‘She involved herself in unsafe sex, that is why she is pregnant.’ Could be maybe she is also exposed to other infections.”

(Kisumu, IDI, Girl 3)

Religious beliefs about adolescent SRH were a barrier to access to services, with many Christian leaders expecting adolescents to abstain from sex. The following excerpt from Kisumu portrays this finding:

“I think the church also thinks family planning is killing.… The Catholic church believes that when you use family planning, you are killing the children that God wanted you to give birth to.”

(Kisumu, IDI, County leader 2)

“A number of religious sects within the county really don’t advocate for the use of contraception and use of reproductive health services. For example, the Legion Maria sect, these exist within the county. Their attitude is that adolescents should abstain. Even the mainstream churches, like Catholics, are pro-life churches and so issues of use of contraception are really prohibited within.”

(Kisumu, IDI, County leader 3)

*Policy level barriers*. Participants mentioned lack of knowledge of ASRH policy as a barrier to ASRH services. This meant that health service providers and teachers were not fully aware of what information and services adolescents were entitled to. Implementation of ASRH policies was lacking in some areas. In addition, the education sector had limitation on health workers’ engagement with adolescents in school settings:

“There is also the lack of awareness on the existence of these policies. How many people know that this policy is in place? Very few people know that these policies exist, and these weakens the enforcement structures.”

(Kisumu, IDI, County leader 1)

“The sooner the national government comes up and rolls out comprehensive sex education in schools, it will give us leverage because now we can go there. And because the education system already allows them, we can get access to the students. But right now, you go and the principal will tell you, ‘No! It is not allowed in schools.”

(Kisumu, IDI, County leader 4)

A lack of resource allocation in county budgets for ASRH meant that program managers and county leaders were limited in the extent to which they could support roll-out of information and services to reach intended beneficiaries

“And I also know that finances are a challenge. As much as we are having the good policies, we know that at times, we need financial assistance to be able to implement this policy, which becomes a challenge.”

*(Kakamega, IDI, County leader 1)*.

“County did not budget enough for the young people in the county budget. This time we have tried again to at least raise the budget. And every year, we have a plan to keep on raising it to enable young people to have a proper allocation on their support.”

(Kisumu, IDI, County leader 1)

#### Facilitators to access and use of ASRH information and services

In this study, there were few ecological model factors that facilitated access to ASRH services.

*Relationship-level facilitators*. Included supportive attitudes by some health workers in some facilities enabled use of ASRH services. For instance, a few adolescent females reported positive experiences with the friendly providers who paid attention to the client-specific needs:

“They are friendly because if you visit them, they handle you according to your sickness.…”

(Kisumu, FGD 2, Girls)

Furthermore, interviews with HCWs illustrated a supportive environment and user-centered approach:

“We [HCWs] allow them to talk freely … to open up. ‘What actually do you want and why do you want it?’ So, we give them an opportunity to make an informed choice. After telling them the consequences and side effects, they say when they are aware, ‘If I use this, this can happen.’”

(Kakamega, IDI, Health Worker 3)

*Organizational-level facilitators*. The organization of health care and its delivery can prompt individuals to seek care. At the health facility, adolescent-reported motivators included short distance to the health facility, availability of free services and supplies, and privacy during service delivery. Some adolescents appreciated when they were given priority in the service queues. This meant that they had shorter waiting time and could get back to school or other chores quickly.

“If you go to the hospital dressed in a [school] uniform, you are attended to first because you are a student and you should go back to class as soon as possible….”

(Kakamega, FGD 3, Boys and Girls)

“There is privacy because anytime you enter the facility, there is a [waiting] room also inside.”

(Kakamega, IDI, Girl 1)

Similarly, at school level, an enabling environment included provision of health insurance to support adolescents to seek health care services was reported. The school reentry policy that allows pregnant girls to continue with their studies until they are due to deliver and are readmitted after giving birth was mentioned as a facilitator, as shown by the quotations below:

“The education act now allows for the pregnant girls to be in school until when they feel they cannot stay longer. So, we allow them to go and deliver and come back after giving birth.”

(Kisumu, IDI, Teacher 2)

“The government is covering students’ health [insurance], so whenever they report to the school that they are unwell, they are given permission to go and seek treatment in the government facilities.”

(Kisumu, IDI, Teacher 3)

*Policy-level facilitators*. Key informants noted that political goodwill had improved, which enabled implementation of ASRH programs and creation of partnerships with other implementing organizations.

“We are seeing that with time, they are beginning to accept that adolescents and sexual reproductive health issues are important and, therefore, when we try to put budget items, we are seeing there is that reduced resistance.”

(Kakamega, IDI, County leader 1)

*“The combined efforts of all organizations working with those issues… makes it easy. When the government wants to go slow, the civil society organizations come in*.

(Kisumu, IDI, County leader 2)”

## Discussion

In this study, we aimed to describe the barriers to and facilitators of access to ASRH services in Kisumu and Kakamega counties, Kenya. We found barriers at all five levels of the ecological model: individual, relationship organization, community, and policy; facilitating factors were found at relationships, organization, and policy. Our findings also show that adolescents in the study areas experienced many health challenges, including sexual and gender-based violence, unprotected sex, drug abuse, girls engaging in sex for money due to poverty, and lack of information and services. Globally, many young people experience violence, which harms their health and dignity and erodes their well-being; for females, much of that violence is perpetuated by intimate partners [20]. These issues have long-term health and social implications for adolescents, including unplanned pregnancy, STIs, and dropping out of school. Progress has been made in the 25 years since the International Conference on Population and Development, which has led to expanded public knowledge about adolescents, their needs and concerns, and ways to help them overcome barriers to SRH services and well-being, and support for fulfilling their aspirations [20]. Despite overall progress in adolescents, progress in many ASRH outcomes has been uneven, both within and between countries, and in some regions, adolescents’ lives have worsened [21]. Kenya has 47 independent counties and the magnitude of ASRH problems varies from one to the other. But Kenya, and other countries, must not only accelerate progress to addressing ASRH challenges that continue to exist, but also create solutions that reduce disparity.

Key barriers at individual level were identified as high cost of services mainly transport costs, consultation fees and medication. Studies in other countries have found similar barriers to access to ASRH services and information. A study in Rwanda on availability, accessibility, and quality of ASRH services found that a majority of adolescents had to travel more than 30 minutes to access SRH services and that only half of the facilities offered low-cost services for adolescents [22]. In Nepal, adolescents were unwilling to visit health facilities because of the lack of availability of SRH items and medicines that are free of cost but are often out of stock, which required adolescents to purchase supplies from private pharmacies [23]. Cost was also a factor in a study conducted in Ethiopia, where adolescents who received drugs during their visit to the health facility were 2.7 times more satisfied and willing to seek services than those who did not [24]. Including cost of ASRH services in county budgets and universal health care packages could help address these individual and organizational barriers.

At the relationship level, studies suggest attitudes of parents are important; a parent who closely participates in their adolescent’s health seeking endeavors can ease the care journey, especially when parental consent is required by law [25]. In addition, our study’s findings imply that health providers with negative attitudes impact uptake of ASRH services. This indicates that more work needs to be done in this area to promote youth-friendly services. This is despite Kenya’s Adolescent Reproductive Health and Development Policy Implementation Assessment, which suggested that government initiatives have resulted in improved attitudes toward ASRH services [26]. Negative and/or judgmental attitudes of health facility staff seem to be a predominant hindrance to uptake of ASRH services in many settings, as reported by a number of research studies [20, 26, 27]. Conversely, we found that supportive health workers facilitated use of ASRH services. Lack of privacy and confidentiality among HCWs was also noted in this and other studies. In a systematic review of 30 studies, eight found that adolescent clients had issues with the lack of privacy and/or confidentiality provided by HCWs [28]. A WHO policy brief on standards for quality ASRH explicitly lists non-judgmental attitudes of staff as part of competency requirements for care providers [29]. Facilities need to provide ASRH services at adolescent-friendly times and set up of youth-friendly corners to minimize missed opportunities for reaching fearful and shy adolescents. There should also be ongoing refresher training to sensitize HCWs on the importance of a positive and friendly attitude, especially for those serving adolescent clients.

At the organization level, availability, accessibility, acceptability, and quality of the ASRH workforce is key in facilitating uptake of services [30]. The shortage of staff (availability) can lead to long queues at the facilities that can hinder uptake of ASRH service, which has been reported by other studies [30]. The most common accessibility barriers identified in past studies were cost of services, the hours the services are offered, and waiting times [31]. Facilitators in this category are convenient opening hours and priority for adolescents in school uniforms when they visited the health facilities. A study of ASRH in Ghana found that adolescents’ concerns are focused on staff friendliness, availability of medication, confidentiality and privacy, and convenient hours of operation [32]. These gaps indicate that Kenya needs to increase access to SRH information to meet global standards for ASRH that were propagated by the WHO on adolescent health literacy [32]. Counties need to ensure availability of an adequate number of trained HCWs, private consultation and testing rooms, and sustained availability of medicines to avoid stock-outs.

In our study, community-level barriers included stigma, religion, myths and misconceptions. Communities shamed adolescents for seeking ASRH services, stigmatizing them for being sexually active while religious leaders believed that if young girls use family planning methods it will negatively affects their reproductive abilities in the future. The disapproval of adolescent sexual activity by community members was also guided by lack of knowledge and their own moral values. Our findings support those from other studies that socio-cultural practices contribute to low levels of use of SRH services. A qualitative systematic review found that low acceptability of ASRH was the most commonly reported barrier to uptake of services in low- and middle-income countries [33]. Stakeholders need to appreciate the realities of adolescents’ lives, including their socio-cultural beliefs and SRH knowledge, and adopt a realistic life-stage approach to providing information and services that are packaged according to the needs within different adolescent age groups. Communities are critical in shaping individual’s behavior and must be involved in ASRH interventions [34].

While key informants noted an improvement in political goodwill, our study revealed that existing policies and legislative frameworks are not adequate to enable ASRH uptake. In addition, these policies were not applied across all levels of care, which creates inequity in access and use of ASRH services [35]. Other countries also face this situation; Shilton et al. reported that although existing laws and policies support the creation of national-level ASRH strategies in Ethiopia, there was a lack of vigorous enforcement and uneven implementation of laws, policies, and strategies [36]. Stakeholders need to ensure that the gap between finalizing and implementing relevant legislation is closed.

### Study strengths and limitations

This study had various strengths. We gained in-depth insights into the experiences of adolescents on seeking SRH services in two counties in Kenya. In addition, the inclusion of a mix of various respondents provided rich information from different stakeholders, which is critical to addressing barriers to ASRH services. Various limitations were also noted. The selection criteria for study participants was purposive and information was self-reported. Both factors may have contributed to over or under reporting and consequently led to information bias. The focus group environment may have introduced social desirability bias and prevented some participants from expressing perspectives that would not be well-accepted by the rest of the group. Data on actual levels of service use were not collected. While Kisumu and Kakamega counties are among those with the high adolescent pregnancy rates in Kenya, the two counties were selected because there was an existing health program in place. Finally, as we selected various participants for FGDs, KIIs, and IDIs, the important category of parents of adolescents as study participants was missed.

## Conclusions and recommendations

We conclude that adolescents continue to face many barriers to access ASRH information and services and recommend that local administration, teachers, parents, and other care providers be sensitized to help adolescents meet their health needs. The school platform is important for delivery of ASRH information and should be strengthened. Counties should strengthen their political will and support dissemination and implementation of existing ASRH policies. Finally, we hope that our findings will enrich project implementation strategies, county planning and positively inform the county leadership teams on ASRH decision-making.

## Supporting information

S1 File (PDF)
