# Supplementary material for: Access to information and use of adolescent sexual reproductive health services: Qualitative exploration of barriers and facilitators in Kisumu and Kakamega, Kenya
Source: PLoS One. 2020 Nov 12;15(11):e0241985. doi: 10.1371/journal.pone.0241985 (PMC7660470; doi:10.1371/journal.pone.0241985)
Supplement: S1 File — (PDF) [file pone.0241985.s001.pdf]

**Access to information and utilization of adolescent sexual reproductive health services: Qualitative exploration of barriers and facilitators in Kisumu and Kakamega, Kenya**

# Interview Guides

## Tool 1: FGD Guide for adolescents

1. What are some of the prevalent health concerns among boys/girls in this community and who do the adolescents discuss with their issues on sexuality?
2. What is your understanding of sexual and reproductive health, what information exists on SRH and what are some of the services that know of?
3. What are some of the services boys and girls typically seek from the healthcare providers, what are their perceptions about services at the local facilities, and what are some of the factors that influence the young people or make it difficult in seeking services?
4. What are some of the myths and beliefs that influence utilization of SRH of services by adolescents' boys and girls, how do the boys and girls make decisions on RH issues, and do the boys and girls receive same treatment when they access services in this community?
5. If you were given an opportunity to design the perfect place for young men and women to receive SRH services, what would this place look like, what are your recommendations to strengthen sexual reproductive health services for adolescents?

## Tool 2: Adolescents IDI guide

1. What are some of things that would encourage/discourage young boys and girls from seeking services at local health facilities?
2. What are some of the challenges that persons of your age face in seeking for reproductive health services?
3. What type of SRH services are available and considered most important and what services are comfortable in accessing from the health facility?
4. Have you ever visited a health facility for SRH services, where did you get the information about services from, and what are some of the socio-cultural taboos that hinder adolescents from accessing information on SRH services?

## Tool 3: IDI guide for Community Representatives

1. What is your understanding of sexual and reproductive health services, and are the services available in the health facility for adolescents, and are there any SRH services that you feel should not be provided to adolescents?
2. What are your views and perceptions about these services and information provided to the adolescents?
3. In your opinion, from where and how do adolescents get information on SRH and when they are in SRH need, from whom or where do they reach out for help?
4. In your opinion, are there any socio cultural practices, customs or taboos that influence the use of sexual and reproductive health services by adolescent girls and boys in this community?
5. In your opinion should parents/caregivers be involved when their adolescents are in need of SRH services, and are there any barriers that make it difficult for boys and girls to access SRH services in this community?

## Tool 4: IDI Guide for Teachers

1. What is your understanding of sexual and reproductive health services, where and how do adolescents get information on SRH, and in your opinion where do the adolescents reach out for help when they are in need for an SRH service?
2. What is your perception of some of the information that is conveyed to adolescents on SRH?
3. Identify socio-cultural factors, taboos and customs that influence adolescent access to sexual and reproductive health information and services.
4. What are some of the services available for adolescents in the health facility, what are some of the reasons that hinder access to these services, are there services that should not be given to the adolescents, should parents be involved in the SRH needs of the adolescents?
5. What is the guidance from ministry of education on provision of ASRH services, and how best can teachers play a role in exploring and documenting the opportunities to increase access to the use of SRH services for adolescents?

## Tool 5: IDI Guide for Health Care Providers

1. What is your understanding of sexual reproductive health services, what services available, what are the greatest worries for the youths in relation to adolescents and do the adolescents get involved in making decisions on their SRH needs and services?
2. What are your views on the utilization of SRH services among adolescents, what would you say are the health needs of the adolescents in this community, and how easy or difficult is it for the adolescents to discuss or talk about sex?
3. What are some of the enabling factors in providing SRH services to adolescents, are their socio cultural barriers to boys and girls accessing services in the facility?
4. What is Current uptake of sexual and reproductive health services by adolescent and youths
5. What are some of the opportunities to increase access and use of sexual reproductive health services by adolescents?

## Tool 6: Key Informant Interviews Guide for County Leaders

1. What are the policies related to SRH information and services among adolescents and youth, and are there barriers and enablers to the implementation of these policies?
2. What are the Sexual and reproductive health needs of adolescents and youths, what are the barriers to the utilization of SRH services by adolescents?
3. What are the myths, misconceptions and perception that influence access to sexual and reproductive health information and services among young people?
4. What are the opportunities and recommendations to improve and strengthen access and use of sexual and reproductive health services by adolescents and youths?
